# Crosstalk between DNA Damage and Inflammation in the Multiple Steps of Carcinogenesis

**DOI:** 10.3390/ijms18081808

**Published:** 2017-08-19

**Authors:** Shosuke Kawanishi, Shiho Ohnishi, Ning Ma, Yusuke Hiraku, Mariko Murata

**Affiliations:** 1 Faculty of Pharmaceutical Sciences, Suzuka University of Medical Science, Suzuka, Mie 513-8670, Japan; shiho-o@suzuka-u.ac.jp; 2 Division of Health Science, Graduate School of Health Science, Suzuka University of Medical Science, Suzuka, Mie 513-8670, Japan; maning@suzuka-u.ac.jp; 3 Department of Environmental and Molecular Medicine, Mie University Graduate School of Medicine, Tsu, Mie 514-8507, Japan; y-hiraku@doc.medic.mie-u.ac.jp (Y.H.); mmurata@doc.medic.mie-u.ac.jp (M.M.)

**Keywords:** oxidative stress, inflammation, cancer

## Abstract

Inflammation can be induced by chronic infection, inflammatory diseases and physicochemical factors. Chronic inflammation is estimated to contribute to approximately 25% of human cancers. Under inflammatory conditions, inflammatory and epithelial cells release reactive oxygen (ROS) and nitrogen species (RNS), which are capable of causing DNA damage, including the formation of 8-oxo-7,8-dihydro-2′-deoxyguanosine and 8-nitroguanine. We reported that 8-nitroguanine was clearly formed at the sites of cancer induced by infectious agents including *Helicobacter pylori*, inflammatory diseases including Barrett’s esophagus, and physicochemical factors including asbestos. DNA damage can lead to mutations and genomic instability if not properly repaired. Moreover, DNA damage response can also induce high mobility group box 1-generating inflammatory microenvironment, which is characterized by hypoxia. Hypoxia induces hypoxia-inducible factor and inducible nitric oxide synthase (iNOS), which increases the levels of intracellular RNS and ROS, resulting DNA damage in progression with poor prognosis. Furthermore, tumor-producing inflammation can induce nuclear factor-κB, resulting in iNOS-dependent DNA damage. Therefore, crosstalk between DNA damage and inflammation may play important roles in cancer development. A proposed mechanism for the crosstalk may explain why aspirin decreases the long-term risk of cancer mortality.

## 1. Introduction

Inflammation can be induced by a wide variety of factors, such as chronic infection, inflammatory diseases and physicochemical agents, and has been recognized to be causative and promotive of cancer [1]. Chronic inflammation is estimated to contribute to approximately 25% of human cancers [2]. Under inflammation, reactive oxygen species (ROS) and nitrogen species (RNS) are produced in inflammatory and epithelial cells, to damage a wide variety of biomolecules including nucleic acids, proteins and lipids [3,4]. ROS and RNS can damage DNA to form mutagenic lesions, such as 8-oxo-7,8-dihydro-2′-deoxyguanosine (8-oxodG) and 8-nitroguanine [5,6,7]. Especially, 8-nitroguanine formation may act as a key molecular event common to various types of inflammation-related carcinogenesis.

Under inflammatory conditions, nitric oxide (NO) is generated in inflammatory and epithelial cells, and this reaction is catalyzed by NO synthase (NOS), especially inducible nitric oxide synthase (iNOS). NO and NOS are known to play roles on both pro- and anti-carcinogenic effects [8]. Sustained induction of iNOS in chronic inflammation can produce ROS and RNS, causing DNA damage [9]. NO reacts with superoxide (O_2_^−^) to form peroxynitrite (ONOO^−^), which causes guanine nitration to generate 8-nitroguanine [10,11]. 8-Nitroguanine is a mutagenic DNA damage. The glycosidic bond between 8-nitroguanine and deoxyribose in DNA strand is thermodynamically unstable. Therefore, 8-nitroguanine can be spontaneously released, leading to the formation of an apurinic site [12]. Adenine can be incorporated opposite the apurinic site during DNA synthesis according to the A-rule. Translesion DNA synthesis, which bypasses apurinic sites, is mediated by DNA polymerase ζ subunits, Rev1 and Rev3 [13]. 8-Nitroguanine can form a pair with adenine during DNA synthesis, and this process is catalyzed by polymerase η and a truncated form of polymerase κ [14]. These suggest that 8-nitroguanine formation leads to single base substitutions, particularly G:C to T:A transversions [15].

**Figure 1:**
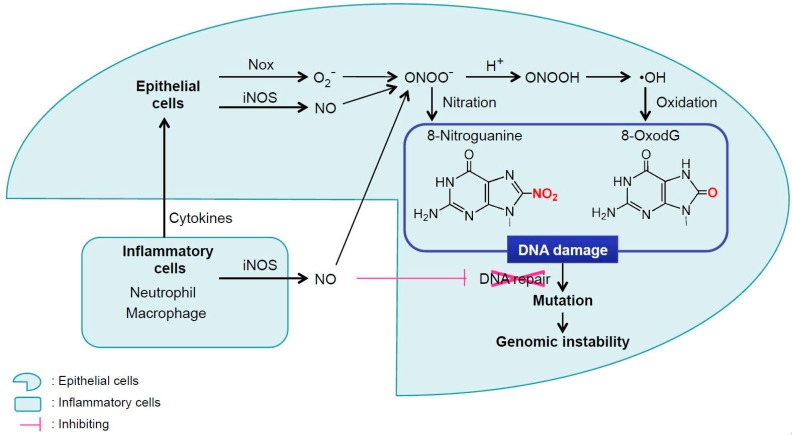
Mechanism for DNA damage in epithelial cells by inflammation. NO and O_2_^−^ are produced during inflammation. Although NO is sufficiently long-lived to diffuse through the extracellular matrix, and enter the nucleus, O_2_^−^ released by neutrophils or macrophages during inflammation is not sufficiently long-lived. Alternatively, inflammatory cells may use cytokines such as TNF-α to stimulate O_2_^−^ formation via Nox in neighboring epithelial cells. NO, which is generated by especially iNOS, reacts with O_2_^−^ forming ONOO^−^, which causes mutagenic DNA damage, such as 8-nitroguanine and 8-oxodG. NO and ROS can participate in inhibition of a number of DNA repair enzymes, which enhances mutations, leading to genomic instability.

NO and O_2_^−^ are released from neutrophils or macrophages during inflammation. NO is long-lived enough to diffuse through the extracellular matrix, cross the plasma membrane and the cytoplasm of epithelial cells, and enter the nucleus, whereas O_2_^−^ is not sufficiently long-lived to react with DNA in the nucleus of epithelial cells [16]. Alternatively, inflammatory cells may release cytokines including tumor necrosis factor α (TNF-α) to stimulate O_2_^−^ accumulation in neighboring epithelial cells [17]. Relevantly, it was reported that interaction of TNF-α and TNF receptor 1 promotes gastric tumorigenesis via the induction of NAD(P)H oxidase (Nox) in tumor cells [18]. Nox generates O_2_^−^ in cancer cells. NO, which is generated by iNOS in tumor-associated macrophage (TAM), reacts with O_2_^−^ in cancer cells, forming ONOO^−^. ONOO^−^ causes DNA damage, mutation and genomic instability to proceed tumor malignancy (Figure 1).

Inflammation increases not only mutagenic DNA lesions, such as 8-nitroguanine and 8-oxodG, but also negatively impacts the DNA repair machinery by inhibiting a number of important DNA repair enzymes [19,20]. For example, *O*-6-methylguanine-DNA methyltransferase (MGMT), the specific DNA repair enzyme, is inhibited by *S*-nitrosation at the reactive and essential cysteine that performs the nucleophilic attack at alkylated nucleobases and confers dealkylation by alkyl transfer, thereby inactivating MGMT itself [19]. DNA damage can lead to mutations and genomic instability if not properly repaired. Genomic instability is defined as higher than normal rates of mutation, which arises from inactivation of DNA repair pathways and high levels of genotoxic ROS and RNS [21]. Therefore, inflammation and the related DNA damage should cause mutation and genomic instability, and finally lead to carcinogenesis. In the following sections, we will review DNA damage under inflammatory condition not only as causative of cancer but also as result of carcinogenesis.

## 2. Inflammation-Mediated DNA Damage

### 2.1 Inflammation-Mediated DNA Damage by Infectious Agents

The International Agency for Research on Cancer (IARC) has estimated that infectious diseases accounts for approximately 18% of cancer cases in the world [22]. We demonstrated that 8-nitroguanine and 8-oxodG were formed in clinical specimens and animal models related to a wide variety of inflammation-related carcinogenesis by immunohistochemical analysis. 8-Nitroguanine formation after iNOS expression was clearly observed in clinical specimens of patients infected with *Helicobacter pylori* (*H. pylori*), hepatitis B virus (HBV), hepatitis C virus (HCV), human papillomavirus (HPV), Epstein-Barr virus (EBV), *Schistosoma haematobium* (SH), and *Opisthorchis viverrini* (OV) [7] (Table 1). IARC has evaluated these infectious agents as group 1 carcinogens (carcinogenic to humans) [22,23]. Interestingly, nitrative and oxidative DNA damage occurred at the sites of carcinogenesis induced by infectious agents and various inflammatory conditions, as reviewed previously [23]. Immunohistochemistry showed that 8-nitroguanine was more clearly stained in the nuclei, when specimens were pre-treated with RNase before immunoreaction. Therefore, 8-nitroguanine was mainly formed in genomic DNA with potent mutagenicity, and might contribute to genomic instability.

### 2.2 Inflammation-Mediated DNA Damage in Inflammatory Diseases

Inflammation-related carcinogenesis can be induced not only by infectious agents, but also by chronic inflammatory diseases (Table 1). Oral diseases such as oral lichen planus (OLP) and leukoplakia, Barrett’s esophagus (BE), and inflammatory bowel diseases (IBDs) are associated with oral squamous cell carcinoma (OSCC), Barrett’s esophageal adenocarcinoma (BEA), and colon cancer, respectively [24,25,26,27,28,29,30].

In oral tissues of leukoplakia patients, histological changes were observed such as epithelial dysplasia and infiltration of inflammatory cells. 8-Nitroguanine and 8-oxodG accumulated in oral epithelium in OLP and OSCC biopsy specimens, whereas staining was not significantly observed in normal oral mucosa [31]. The accumulation of DNA damage was related to the expression of iNOS and the accumulation of 3-nitrotyrosine, an indicator of nitrative stress. An accumulation of mutated p53 was also observed in oral epithelium, more strongly in OSCC than in OLP, whereas p53 accumulation was not observed in normal oral mucosa. These suggest that iNOS-dependent DNA damage may lead to aberrant p53 accumulation and participates in oral carcinogenesis by OLP.

In mouse model for IBDs, we demonstrated that accumulations of 8-nitroguanine and 8-oxodG in colon epithelial cells [32]. This model showed severe inflammation in colon tissues and similar pathological findings to those of IBDs patients. The accumulation of DNA lesions was related to the expression of iNOS, proliferating cell nuclear antigen, and p53. These suggest that iNOS-dependent DNA damage is induced in colon epithelial cells of IBD model mice and may lead to cell proliferation and colon carcinogenesis.

In relation to BE patients, we observed that levels of 8-nitroguanine, 8-oxodG and iNOS were significantly higher in the order of BEA > BE > normal tissues [33,34]. In case of proton pump inhibitors (PPIs) treatment, which is expected to reduce BEA risk, DNA damage was significantly decreased in BE tissues. Moreover, the expression of Mn-SOD, an antioxidant enzyme, and the nuclear localization of Nrf2, the transcription factor of Mn-SOD, were significantly increased in BE tissues after PPIs treatment. These suggest that 8-nitroguanine and 8-oxodG play a role in BE-derived carcinogenesis, and that these DNA lesions may be suppressed by PPIs treatment not only by reduction of gastric acid, but also by activation of Nrf2 resulting in the expression of the antioxidant enzyme Mn-SOD.

### 2.3 Inflammation-Mediated DNA Damage by Particulate Matters

Chronic inflammation can be induced by many physical, chemical and immunological factors [35]. Inhalation of particulate matters, including diesel engine exhaust and nanomaterials, causes chronic inflammation in respiratory systems, that may lead to chronic obstructive pulmonary disease and cancer [36,37,38,39,40]. We reported that nitrative DNA damage was strongly formed at related cancer sites by particulate matters such as asbestos [41,42].

Asbestos is mineral fiber, which has been used as heat insulting material. It causes lung cancer and malignant mesothelioma in humans [43]. In asbestos-exposed mice, 8-nitroguanine was formed in the nucleus of bronchial epithelial cells, whereas 8-nitroguanine formation was not significantly observed in control mice [41]. In humans, 8-nitroguanine formation was associated with asbestos contents in lung tissues [42]. The precise mechanisms of asbestos-induced carcinogenesis have not well been understood, but appear to involve the following molecular events: (a) irritation of the tissues; (b) severing and/or piercing the mitotic spindle, resulting in disruption of mitosis and chromosomal damage including aneuploidy; (c) ROS generation catalyzed by iron to cause DNA damage [44].

Carbon nanotube (CNT) is an allotrope of carbon with a cylindrical shape, and expected to be used in the field of material science because of its unique physicochemical property [45,46]. However, intraperitoneal and intrascrotal administration of CNT caused mesothelioma in experimental animals, possibly involving chronic inflammation [47,48,49,50]. We have demonstrated that multi-walled CNT is taken up into cells through clathrin- and caveolae-mediated endocytosis, leading to inflammatory responses including iNOS expression and 8-nitroguanine formation, by using A549 human lung epithelial cells [51,52].

Carbon black (CB) is an extremely fluffy fine powder composed, which is widely used as a pigment in tires, inks, paints, coatings, and plastics [53]. Inhalation exposure of CB causes malignant lung tumors in experimental animals [40]. Our study revealed that CB induced 8-nitroguanine formation mainly in the nucleus of cells, after internalization into RAW 264.7 macrophage and A549 lung epithelial cells by clathrin-mediated endocytosis [54]. This finding raises a possibility that RNS released from CB-exposed inflammatory cells may also cause DNA damage in adjacent epithelial cells, contributing to carcinogenesis.

## 3. DNA Damage and Inflammation Interplay

### 3.1 Hypoxia-Related DNA Damage and Prognosis

Cancer is a disease potentiated by a great number of mutations in somatic cells. Using the integrated data sets, Kandoth et al. identified 127 significantly mutated genes in cancer [55,56]. Mutations are produced by not only DNA damage by environmental factors but also DNA damage mediated by inflammatory molecules of endogenous origin. Inflammatory microenvironment tends to hypoxia.

Intratumoral hypoxia induces a rapid increase in the expression of hypoxia-inducible factor (HIF)-1 protein, a heterodimer consisting of α and β subunits. Under hypoxic conditions, HIF-1α degradation is suppressed and this subunit dimerizes with HIF-1β to form the heterodimer in the nucleus, which promotes the expression of numerous target genes, including iNOS. We observed clear 8-nitroguanine formation in the tumor cells and inflammatory cells in patients with malignant fibrous histiocytoma (MFH), while HIF-1α expression was observed in the tumor cells [57]. The Kaplan-Meier method revealed that survival curves significantly differed between the groups of high and low 8-nitroguanine staining as well as HIF-1α [57].

MFH, which is one of the most common soft tissue sarcomas, is considered to be a lesion associated with inflammatory responses. We have examined nitrative DNA damage in tumor tissues of MFH patients by immunohistochemical analyses and the association with the prognosis [58]. Staining intensities of 8-nitroguanine and 8-oxodG were much greater in MFH tissues of deceased patients than in live patients. Interestingly, survival curves analyzed by the Kaplan-Meier method showed that the staining intensity of 8-nitroguanine was significantly associated with poor prognosis. These results suggest that 8-nitroguanine participates in not only initiation but also progression and conversion of MFH, and could be a potential biomarker to evaluate the prognosis of cancer patients.

These results and the previous study [58] suggest that iNOS-dependent 8-nitroguanine formation by HIF-1α and nuclear factor-κB (NF-κB) plays a role in tumor progression and conversion [57].

Chronic infection with the liver fluke OV is closely associated with the pathogenesis of cholangiocarcinoma. We examined the relationship of DNA lesions and HIF-1α expression with tumor invasion in intrahepatic cholangiocarcinoma patients [59]. Immunohistochemical analysis showed that 8-nitroguanine and 8-oxodG were formed to a much greater extent in tumor tissues than in non-tumor tissues. HIF-1α expression was detected in tumor tissues in all patients, and correlated with iNOS expression and 8-oxodG formation. Double immunofluorescence technique revealed that iNOS and HIF-1α were co-localized in tumor tissues. Notably, 8-oxodG formation was significantly associated with lymphatic invasion. Moreover, 8-nitroguanine and 8-oxodG formation in non-tumor tissues were associated with neural invasion. These results raise a possibility that HIF-1α and iNOS activate each other to mediate persistent DNA damage, leading to accumulation of mutation, acquiring tumor invasiveness and poor prognosis.

### 3.2 Tumor Microenvironment-Induced Inflammation Followed by DNA Damage

Solid tumors often show signs of inflammation [60] and recruit innate immune cells, such as macrophages. TAM can constitutes up to 50% of the tumor mass [61,62]. TAMs accelerate progression by cytokines such as interleukin-6 (IL-6), which induces signal transducer and activator of transcription (STAT) 3 signaling, to promote growth, invasion and metastasis [63].

Nasopharyngeal carcinoma (NPC) is closely associated with EBV infection. We performed immunofluorescent staining to examine DNA damage in biopsy and surgical specimens of nasopharyngeal tissues from NPC patients in southern China [64]. Strong DNA damage occurred in cancer cells and inflammatory cells in the stroma of NPC patients. Intensive iNOS expression was observed in the cytoplasm of cancer cells positive for 8-nitroguanine. Staining of DNA lesions and iNOS was observed in epithelial cells of EBV-positive chronic nasopharyngitis patients as well, although their staining intensities were weaker than those in NPC patients. In EBV-negative subjects, no or weak staining of DNA lesions and iNOS was observed. EGFR and phosphorylated STAT3 were clearly expressed in cancer cells of NPC patients, whereas no or weak NF-κB expression was induced. This result suggests that STAT3-dependent signaling pathway plays an important role in NPC carcinogenesis rather than NF-κB-mediated pathway. IL-6 was expressed mainly in macrophages present in nasopharyngeal tissues of patients with EBV infection. An in vitro experiment showed that IL-6 induced the expression of phosphorylated STAT3 and iNOS. These results suggest that EGFR accumulation in the nucleus and IL-6-mediated STAT3 activation participate in nitrative DNA damage, leading to EBV-mediated carcinogenesis.

Hypoxia is a characteristic feature of both tumors and inflammation [63]. Hypoxia promotes translocation of high mobility group box 1 (HMGB1) from the nucleus to the cytoplasm [65]. Extracellular HMGB1 promotes proliferation, inflammation, and angiogenesis and inhibits host anticancer immunity, which contributes to tumorigenesis [66]. In our study, CNT significantly increased nitrative DNA damage in A549 human lung epithelial cells through the release of HMGB1 and DNA into the extracellular space. HMGB1 and DNA forms a complex, which binds to receptor for advanced glycation end products on neighboring cells, and CpG DNA is recognized by Toll-like receptor (TLR) 9 in lysosomes, leading to NO generation and 8-nitroguanine formation [51,52].

In cancer cells, immunogenic cell death, which is induced by ROS and RNS, can induce HMGB1 expression [67]. HMGB1 is passively released from damaged cells or necrotic cells [68]. Extracellular HMGB1 promotes NF-κB transportation to the nucleus and induces the expression of inflammatory molecules and tumor cell proliferation through TLR4-dependent pathway [69,70]. The mechanism of tumor progression is associated with local and chronic persistent inflammation. HMGB1 induces both recruited leukocytes and settled immune cells to release cytokines including TNF-α and IL-6, which amplify and extend the inflammatory response [71,72].

NF-κB is a key player in inflammation and regulates iNOS expression [73,74]. TNF-α and IL-6 activate each other to form the cytokine network in tumor tissues [75]. These cytokines are shown to induce iNOS expression, resulting in the formation of mutagenic DNA lesions and carcinogenesis under inflammatory microenvironment [73,74,76,77,78]. In this paper, the mechanism tends to focus only NF-κB and STAT3, although there are several papers suggesting the role of other signals on carcinogenesis. For example, mitogen-activated protein kinasess are also important in the process of inflammation and cancer [79].

In response to ROS and DNA damage, autophagy is activated as well as low levels of cellular nutrients. Autophagy is required for several functional outcomes of DNA damage response (DDR) signaling, including repair of DNA lesions, and cell death. If not repaired, DNA damage may result in cell death and also be a major source of genomic instability. Recent studies have provided evidence showing that DDR and immune response networks functionally interact and that autophagy is involved in regulation of inflammatory pathways [80,81]. In summary, DNA damage events trigger the activation of DDR-driven pro-inflammatory signals, including NF-κB or various interleukins leading to chronic inflammation.

## 4. Expectation of Non-Steroidal Anti-Inflammatory Drugs (NSAIDs) as Chemical Cancer Prevention

NO can stimulate tumor growth and metastasis by promoting migration and invasion of tumor cells and angiogenesis, which can be triggered by cyclooxygenase-2 (COX-2) activation. Thus, selective inhibitors of NOS and/or COX may have a therapeutic potential against various cancers [9].

**Figure 2:**
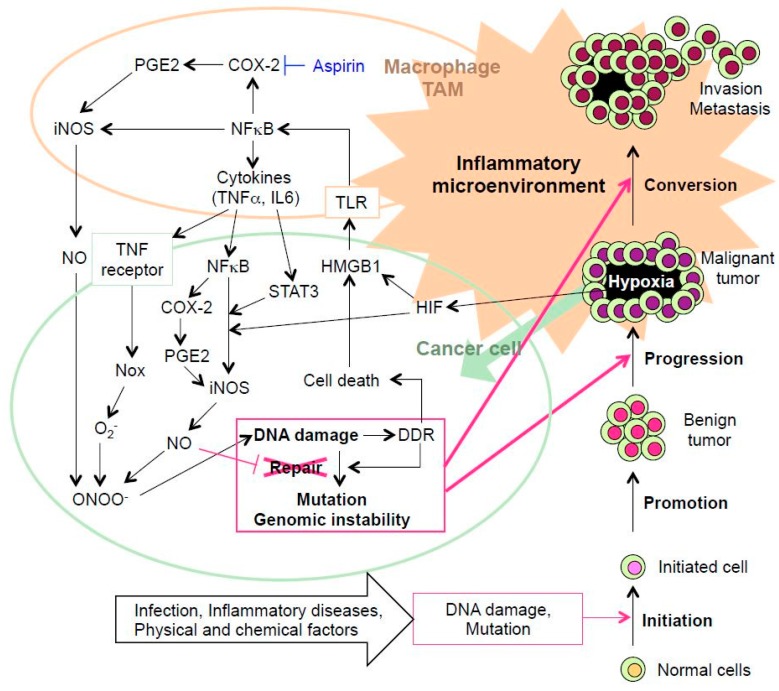
Mechanism for crosstalk between DNA damage and inflammation in the multiple steps of carcinogenesis. In cancer cells, cell death, which is induced by ROS and RNS, can induce HMGB1 expression. HMGB1 is passively released into the extracellular space from damaged or necrotic cells. Hypoxia induces HIF-1 expression, which regulates iNOS expression, and promotes translocation of HMGB1 from the nucleus to the cytoplasm. Via TLR4, extracellular HMGB1 can promote NF-κB transportation to the nucleus and induce the expression of TNF-α and IL-6, which induce iNOS expression. NO, which is generated by iNOS in tumor-associated macrophage (TAM), is released to the extracellular space and interacts with O_2_^−^ generated via Nox in cancer cells to form ONOO^−^. ONOO^−^ causes DNA damage resulting in mutations and genomic instability as not properly repaired. NF-κB induces not only iNOS but also COX-2 expression. COX-2 participates in formation of PGE2, which induces iNOS. Aspirin can inhibit COX-2, resulting in the decrease of PGE2 and iNOS and suppression of the crosstalk between DNA damage and inflammation in cancer development. This is a possible mechanism by which aspirin reduces the long-term risk of death due to cancer.

Several papers have revealed that daily aspirin, one of NSAIDs, reduces the long-term risk of cancer mortality [82,83,84,85,86,87,88]. Reduction in cancer mortality has been shown in colon cancer, probably in prostate cancer and possibly in breast cancer [87]. Rothwell et al. reported that aspirin of 5 years or longer reduced about 70% of risk of proximal colon cancer [88]. Meta-analyses have demonstrated that low-dose aspirin reduces the risk of metastasis of colon adenocarcinoma by 83% [89]. The present review has supported that NSAIDs such as aspirin show prevent effect on cancer. In inflammatory pathways, the signaling cascade causes the translocation of NF-κB in to the nucleus, where it induces pro-inflammatory genes including iNOS and COX-2. COX-2 catalyzes the conversion of arachidonic acid to prostaglandin H2 (PGH2), which is further converted into prostaglandin E2 (PGE2) by terminal prostaglandin Esynthase. PGE2 transduces signals via four different G-protein coupled receptors [90]. The prolonged release of PGE2 stimulates receptors on macrophages to induce iNOS through adenylyl cyclase/cyclic AMP/extracellular signal-regulated kinase signal [91]. Aspirin can inhibit COX-2, resulting in the decrease of PGE2 and iNOS, which should suppress the crosstalk between DNA damage and inflammation in cancer development (Figure 2). This is a possible mechanism by which aspirin reduces the long-term risk of cancer mortality.

## 5. Conclusions

Infectious agents such as *H. pylori*, inflammatory diseases such as BE, and physicochemical factors, such as asbestos, cause DNA damage via chronic inflammation, leading to mutation and genomic instability. The initiated cell, which can escape from apoptosis, proliferates to form benign tumor. Inflammation is important causative of cancer and is promoted in cancer microenvironment including TAMs. In inflammatory microenvironment, DNA damage and mutation are accumulated, leading to carcinogenesis via genomic instability. Moreover, DNA damage response can also induce inflammation, again. Therefore, crosstalk between DNA damage and inflammation may play important roles in cancer development. DNA damage initiated from inflammation appears to be a key tool for the inflammation to transition to the cancer, and this helps understand the long-standing concept that inflammation is one of precancerous symptoms.

## Figures and Tables

**Table 1:** Causative factors and cancer sites, in which 8-nitroguanine, inflammation-related DNA damage, accumulated.

Causative Factors			Cancer Sites
Infectious agents	Bacteria	*H. pylori*	Stomach
	Viruses	HPV	Cervix and other sites
		HBV	Liver
		HCV	
		EBV	Lymph node, nasopharynx and other sites
	Parasites	SH	Bladder
		OV	Bile duct
Inflammatory diseases	OLP		Oral cavity
	BE		Esophagus
	IBDs		Colon
	MFH		Soft tissue
Particulate matters	Asbestos		Mesothelium, lung

## Abbreviations

8-oxodG	8-oxo-7,8-dihydro-2’-deoxyguanosine
BE	Barrett’s esophagus
BEA	Barrett’s esophageal adenocarcinoma
CB	Carbon black
CNT	Carbon nanotube
COX-2	Cyclooxygenase 2
DDR	DNA damage response
EBV	Epstein-Barr virus
*H. pylori*	*Helicobacter pylori*
HBV	Hepatitis B virus
HCV	Hepatitis C virus
HMGB1	High mobility group box 1
HPV	Human papillomavirus
HIF	Hypoxia-inducible factor
iNOS	Inducible nitric oxide synthase
IBDs	Inflammatory bowel diseases
IL-6	Interleukin-6
IARC	International Agency for Research on Cancer
MFH	Malignant fibrous histiocytoma
Nox	NAD(P)H oxidase
NO	Nitric oxide
NOS	NO synthase
NSAIDs	Non-steroidal anti-inflammatory drugs
NF-κB	Nuclear factor-κB
OLP	Oral lichen planus
OSCC	Oral squamous cell carcinoma
OV	*Opisthorchis viverrini*
ONOO^−^	Peroxynitrite
RNS	Reactive nitrogen species
PGH2	Prostaglandin H2
PGE2	Prostaglandin E2
PPIs	Proton pump inhibitors
ROS	Reactive oxygen species
SH	*Schistosoma haematobium*
STAT	Signal transducer and activator of transcription
O_2_^−^	Superoxide
TNF-α	Tumor necrosis factor alpha
TAM	Tumor-associated macrophage
TLR	Toll-like receptor

## References

[1] Coussens L.M., Werb Z. (2002). Inflammation and cancer. Nature.

[2] Hussain S.P., Harris C.C. (2007). Inflammation and cancer: An ancient link with novel potentials. Int. J. Cancer.

[3] Hussain S.P., Hofseth L.J., Harris C.C. (2003). Radical causes of cancer. Nat. Rev. Cancer.

[4] Wiseman H., Halliwell B. (1996). Damage to DNA by reactive oxygen and nitrogen species: Role in inflammatory disease and progression to cancer. Biochem. J..

[5] Kawanishi S., Hiraku Y. (2006). Oxidative and nitrative DNA damage as biomarker for carcinogenesis with special reference to inflammation. Antioxid. Redox Signal..

[6] Murata M., Thanan R., Ma N., Kawanishi S. (2012). Role of nitrative and oxidative DNA damage in inflammation-related carcinogenesis. J. Biomed. Biotechnol..

[7] Kawanishi S., Ohnishi S., Ma N., Hiraku Y., Oikawa S., Murata M. (2017). Nitrative and oxidative DNA damage in infection-related carcinogenesis in relation to cancer stem cells. Genes Environ..

[8] Fukumura D., Kashiwagi S., Jain R.K. (2006). The role of nitric oxide in tumour progression. Nat. Rev. Cancer.

[9] Lala P.K., Chakraborty C. (2001). Role of nitric oxide in carcinogenesis and tumour progression. Lancet Oncol..

[10] Halliwell B. (1999). Oxygen and nitrogen are pro-carcinogens. Damage to DNA by reactive oxygen, chlorine and nitrogen species: Measurement, mechanism and the effects of nutrition. Mutat. Res..

[11] Sodum R.S., Fiala E.S. (2001). Analysis of peroxynitrite reactions with guanine, xanthine, and adenine nucleosides by high-pressure liquid chromatography with electrochemical detection: C8-nitration and -oxidation. Chem. Res. Toxicol..

[12] Yermilov V., Rubio J., Ohshima H. (1995). Formation of 8-nitroguanine in DNA treated with peroxynitrite in vitro and its rapid removal from DNA by depurination. FEBS Lett..

[13] Wu X., Takenaka K., Sonoda E., Hochegger H., Kawanishi S., Kawamoto T., Takeda S., Yamazoe M. (2006). Critical roles for polymerase ζ in cellular tolerance to nitric oxide-induced DNA damage. Cancer Res..

[14] Suzuki N., Yasui M., Geacintov N.E., Shafirovich V., Shibutani S. (2005). Miscoding events during DNA synthesis past the nitration-damaged base 8-nitroguanine. Biochemistry.

[15] Loeb L.A., Preston B.D. (1986). Mutagenesis by apurinic/apyrimidinic sites. Annu. Rev. Genet..

[16] Carballal S., Bartesaghi S., Radi R. (2014). Kinetic and mechanistic considerations to assess the biological fate of peroxynitrite. Biochim. Biophys. Acta.

[17] Grivennikov S.I., Greten F.R., Karin M. (2010). Immunity, inflammation, and cancer. Cell.

[18] Oshima H., Ishikawa T., Yoshida G.J., Naoi K., Maeda Y., Naka K., Ju X., Yamada Y., Minamoto T., Mukaida N. (2014). TNF-α/TNFR1 signaling promotes gastric tumorigenesis through induction of Noxo1 and Gna14 in tumor cells. Oncogene.

[19] Mikhed Y., Görlach A., Knaus U.G., Daiber A. (2015). Redox regulation of genome stability by effects on gene expression, epigenetic pathways and DNA damage/repair. Redox Biol..

[20] Moritz E., Pauly K., Bravard A., Hall J., Radicella J.P., Epe B. (2014). hOGG1-Cys326 variant cells are hypersensitive to DNA repair inhibition by nitric oxide. Carcinogenesis.

[21] Tubbs A., Nussenzweig A. (2017). Endogenous DNA damage as a source of genomic instability in cancer. Cell.

[22] Boyle P., Levin B., IARC (2008). Chronic infections. World Cancer Report.

[23] IARC (2009). Opisthorchis viverrini and clonorchis sinensis. IARC Monographs on the Evaluation of Carcinogenic Risks to Humans.

[24] Podolsky D.K. (2002). Inflammatory bowel disease. N. Engl. J. Med..

[25] Ekbom A., Helmick C., Zack M., Adami H.O. (1990). Increased risk of large-bowel cancer in Crohn’s disease with colonic involvement. Lancet.

[26] Langholz E., Munkholm P., Davidsen M., Binder V. (1992). Colorectal cancer risk and mortality in patients with ulcerative colitis. Gastroenterology.

[27] Choi P.M., Zelig M.P. (1994). Similarity of colorectal cancer in Crohn’s disease and ulcerative colitis: implications for carcinogenesis and prevention. Gut.

[28] Wild C.P., Hardie L.J. (2003). Reflux, Barrett’s oesophagus and adenocarcinoma: Burning questions. Nat. Rev. Cancer.

[29] Rajentheran R., McLean N.R., Kelly C.G., Reed M.F., Nolan A. (1999). Malignant transformation of oral lichen planus. Eur. J. Surg. Oncol..

[30] Mignogna M.D., Fedele S., lo Russo L., lo Muzio L., Bucci E. (2004). Immune activation and chronic inflammation as the cause of malignancy in oral lichen planus: Is there any evidence?. Oral Oncol..

[31] Chaiyarit P., Ma N., Hiraku Y., Pinlaor S., Yongvanit P., Jintakanon D., Murata M., Oikawa S., Kawanishi S. (2005). Nitrative and oxidative DNA damage in oral lichen planus in relation to human oral carcinogenesis. Cancer Sci..

[32] Ding X., Hiraku Y., Ma N., Kato T., Saito K., Nagahama M., Semba R., Kuribayashi K., Kawanishi S. (2005). Inducible nitric oxide synthase-dependent DNA damage in mouse model of inflammatory bowel disease. Cancer Sci..

[33] Thanan R., Ma N., Hiraku Y., Iijima K., Koike T., Shimosegawa T., Murata M., Kawanishi S. (2016). DNA damage in CD133-positive cells in Barrett’s esophagus and esophageal adenocarcinoma. Mediat. Inflamm..

[34] Thanan R., Ma N., Iijima K., Abe Y., Koike T., Shimosegawa T., Pinlaor S., Hiraku Y., Oikawa S., Murata M., Kawanishi S. (2012). Proton pump inhibitors suppress iNOS-dependent DNA damage in Barrett's esophagus by increasing Mn-SOD expression. Biochem. Biophys. Res. Commun..

[35] Ohshima H., Tatemichi M., Sawa T. (2003). Chemical basis of inflammation-induced carcinogenesis. Arch. Biochem. Biophys..

[36] Wong J., Magun B.E., Wood L.J. (2016). Lung inflammation caused by inhaled toxicants: A review. Int. J. Chron. Obstruct. Pulmon. Dis..

[37] Jacobsen N.R., Moller P., Jensen K.A., Vogel U., Ladefoged O., Loft S., Wallin H. (2009). Lung inflammation and genotoxicity following pulmonary exposure to nanoparticles in ApoE^-/-^ mice. Part. Fibre Toxicol..

[38] Porter D.W., Hubbs A.F., Mercer R.R., Wu N., Wolfarth M.G., Sriram K., Leonard S., Battelli L., Schwegler-Berry D., Friend S. (2010). Mouse pulmonary dose- and time course-responses induced by exposure to multi-walled carbon nanotubes. Toxicology.

[39] Shvedova A.A., Kisin E.R., Porter D., Schulte P., Kagan V.E., Fadeel B., Castranova V. (2009). Mechanisms of pulmonary toxicity and medical applications of carbon nanotubes: Two faces of Janus?. Pharmacol. Ther..

[40] Heinrich U., Fuhst R., Rittinghausen S., Creutzenberg O., Bellmann B., Koch W., Levsen K. (1995). Chronic inhalation exposure of Wistar rats and two different strains of mice to diesel engine exhaust carbon black, and titanium dioxide. Inhal. Toxicol..

[41] Hiraku Y., Kawanishi S., Ichinose T., Murata M. (2010). The role of iNOS-mediated DNA damage in infection- and asbestos-induced carcinogenesis. Ann. N. Y. Acad. Sci..

[42] Hiraku Y., Sakai K., Shibata E., Kamijima M., Hisanaga N., Ma N., Kawanishi S., Murata M. (2014). Formation of the nitrative DNA lesion 8-nitroguanine is associated with asbestos contents in human lung tissues: A pilot study. J. Occup. Health.

[43] IARC (2009). Asbestos (chrysotile, amosite, crocidolite, tremolite, actinolite, and anthophyllite). IARC Monographs on the Evaluation of Carcinogenic Risks to Humans.

[44] Robinson B.W., Lake R.A. (2005). advances in malignant mesothelioma. N. Engl. J. Med..

[45] Medina C., Santos-Martinez M.J., Radomski A., Corrigan O.I., Radomski M.W. (2007). Nanoparticles: Pharmacological and toxicological significance. Br. J. Pharmacol..

[46] Pacurari M., Yin X.J., Zhao J., Ding M., Leonard S.S., Schwegler-Berry D., Ducatman B.S., Sbarra D., Hoover M.D., Castranova V. (2008). Raw single-wall carbon nanotubes induce oxidative stress and activate MAPKs, AP-1, NF-κB, and Akt in normal and malignant human mesothelial cells. Environ. Health Perspect..

[47] Nagai H., Okazaki Y., Chew S.H., Misawa N., Yamashita Y., Akatsuka S., Ishihara T., Yamashita K., Yoshikawa Y., Yasui H. (2011). Diameter and rigid- ity of multiwalled carbon nanotubes are critical factors in mesothelial injury and carcinogenesis. Proc. Natl. Acad. Sci. USA.

[48] Poland C.A., Duffin R., Kinloch I., Maynard A., Wallace W.A., Seaton A., Stone V., Brown S., MacNee W., Donaldson K. (2008). Carbon nanotubes introduced into the abdominal cavity of mice show asbestos-like pathogenicity in a pilot study. Nat. Nanotechnol..

[49] Takagi A., Hirose A., Nishimura T., Fukumori N., Ogata A., Ohashi N., Kitajima S., Kanno J. (2008). Induction of mesothelioma in p53^+/-^ mouse by intraperitoneal application of multi-wall carbon nanotube. J. Toxicol. Sci..

[50] Sakamoto Y., Nakae D., Fukumori N., Tayama K., Maekawa A., Imai K., Hirose A., Nishimura T., Ohashi N., Ogata A. (2009). Induction of mesothelioma by a single intrascrotal administration of multi-wall carbon nanotube in intact male Fischer 344 rats. J. Toxicol. Sci..

[51] Guo F., Ma N., Horibe Y., Kawanishi S., Murata M., Hiraku Y. (2012). Nitrative DNA damage induced by multi-walled carbon nanotube via endocytosis in human lung epithelial cells. Toxicol. Appl. Pharmacol..

[52] Hiraku Y., Guo F., Ma N., Yamada T., Wang S., Kawanishi S., Murata M. (2016). Multi-walled carbon nanotube induces nitrative DNA damage in human lung epithelial cells via HMGB1-RAGE interaction and Toll-like receptor 9 activation. Part. Fibre Toxicol..

[53] IARC (2010). Carbon black, titanium dioxide, and talc. IARC Monographs on the Evaluation of Carcinogenic Risks to Humans.

[54] Hiraku Y., Nishikawa Y., Ma N., Afroz T., Mizobuchi K., Ishiyama R., Matsunaga Y., Ichinose T., Kawanishi S., Murata M. (2017). Nitrative DNA damage induced by carbon-black nanoparticles in macrophages and lung epithelial cells. Mutat. Res..

[55] Kandoth C., McLellan M.D., Vandin F., Ye K., Niu B., Lu C., Xie M., Zhang Q., McMichael J.F., Wyczalkowski M.A. (2013). Mutational landscape and significance across 12 major cancer types. Nature.

[56] Loeb L.A. (2011). Human cancers express mutator phenotypes: Origin, consequences and targeting. Nat. Rev. Cancer.

[57] Hoki Y., Murata M., Hiraku Y., Ma N., Matsumine A., Uchid A., Kawanishi S. (2007). 8-Nitroguanine as a potential biomarker for progression of malignant fibrous histiocytoma, a model of inflammation-related cancer. Oncol. Rep..

[58] Hoki Y., Hiraku Y., Ma N., Murata M., Matsumine A., Nagahama M., Shintani K., Uchid A., Kawanishi S. (2007). iNOS-dependent DNA damage in patients with malignant fibrous histiocytoma in relation to prognosis. Cancer Sci..

[59] Pinlaor S., Sripa B., Ma N., Hiraku Y., Yongvanit P., Wongkham S., Pairojkul C., Bhudhisawasdi V., Oikawa S., Murata M. (2005). Nitrative and oxidative DNA damage in intrahepatic cholangiocarcinoma patients in relation to tumor invasion. World J. Gastroenterol..

[60] Carvalho M.I., Silva-Carvalho R., Pires I., Prada J., Bianchini R., Jensen-Jarolim E., Queiroga F.L. (2016). A comparative approach of tumor-associated inflammation in mammary cancer between humans and dogs. BioMed Res. Int..

[61] Mamlouk S., Wielockx B. (2013). Hypoxia-inducible factors as key regulators of tumor inflammation. Int. J. Cancer.

[62] Guo Q., Jin Z., Yuan Y., Liu R., Xu T., Wei H., Xu X., He S., Chen S., Shi Z. (2016). New mechanisms of tumor-associated macrophages on promoting tumor progression: Recent research advances and potential targets for tumor immunotherapy. J. Immunol. Res..

[63] Triner D., Shah Y.M. (2016). Hypoxia-inducible factors: A central link between inflammation and cancer. J. Clin. Investig..

[64] Ma N., Kawanishi M., Hiraku Y., Murata M., Huang G.W., Huang Y., Luo D.Z., Mo W.G., Fukui Y., Kawanishi S. (2008). Reactive nitrogen species-dependent DNA damage in EBV-associated nasopharyngeal carcinoma: The relation to STAT3 activation and EGFR expression. Int. J. Cancer.

[65] Pistoia V., Pezzolo A. (2016). Involvement of HMGB1 in resistance to tumor vessel-targeted, monoclonal antibody-based immunotherapy. J. Immunol. Res..

[66] Kang R., Zhang Q., Zeh H.J., Lotze M.T., Tang D. (2013). HMGB1 in cancer: Good, bad, or both?. Clin. Cancer Res..

[67] Shalapour S., Karin M. (2015). Immunity, inflammation, and cancer: An eternal fight between good and evil. J. Clin. Investig..

[68] He S.J., Cheng J., Feng X., Yu Y., Tian L., Huang Q. (2017). The dual role and therapeutic potential of high-mobility group box 1 in cancer. Oncotarget.

[69] Tadie J.M., Bae H.B., Deshane J.S., Bell C.P., Lazarowski E.R., Chaplin D.D., Thannickal V.J., Abraham E., Zmijewski J.W. (2012). Toll-like receptor 4 engagement inhibits adenosine 5′-monophosphate-activated protein kinase activation through a high mobility group box 1 protein-dependent mechanism. Mol. Med..

[70] Weng H., Deng Y., Xie Y., Liu H., Gong F. (2013). Expression and significance of HMGB1, TLR4 and NF-κB p65 in human epidermal tumors. BMC Cancer.

[71] Jube S., Rivera Z.S., Bianchi M.E., Powers A., Wang E., Pagano I., Pass H.I., Gaudino G., Carbone M., Yang H. (2012). Cancer cell secretion of the DAMP protein HMGB1 supports progression in malignant mesothelioma. Cancer Res..

[72] Yang H., Tracey K.J. (2010). Targeting HMGB1 in inflammation. Biochim. Biophys. Acta.

[73] Surh Y.J., Chun K.S., Cha H.H., Han S.S., Keum Y.S., Park K.K., Lee S.S. (2001). Molecular mechanisms underlying chemopreventive activities of anti- inflammatory phytochemicals: Down-regulation of COX-2 and iNOS through suppression of NF-κB activation. Mutat. Res..

[74] Balkwill F., Coussens L.M. (2004). Cancer: An inflammatory link. Nature.

[75] Echizen K., Hirose O., Maeda Y., Oshima M. (2016). Inflammation in gastric cancer: Interplay of the COX-2/prostaglandin E2 and Toll-like receptor/MyD88 pathways. Cancer Sci..

[76] Vannini F., Kashfi K., Nath N. (2015). The dual role of iNOS in cancer. Redox Biol..

[77] Nathan C. (1992). Nitric oxide as a secretory product of mammalian cells. FASEB J..

[78] Ferreiro C.R., Chagas A.C., Carvalho M.H., Dantas A.P., Jatene M.B., Bento de Souza L.C., Lemos da Luz P. (2001). Influence of hypoxia on nitric oxide synthase activity and gene expression in children with congenital heart disease: A novel pathophysiological adaptive mechanism. Circulation.

[79] Gupta J., Nebreda A.R. (2015). Roles of p38α mitogen-activated protein kinase in mouse models of inflammatory diseases and cancer. FEBS J..

[80] Eliopoulos A.G., Havaki S., Gorgoulis V.G. (2016). DNA damage response and autophagy: A meaningful partnership. Front. Genet..

[81] Moreno-Villanueva M., Bürkle A. (2016). Stress hormone-mediated DNA damage response--Implications for cellular senescence and tumour progression. Curr. Drug Targets.

[82] Thun M.J., Jacobs E.J., Patrono C. (2012). The role of aspirin in cancer prevention. Nat. Rev. Clin. Oncol..

[83] Jacobs E.J., Newton C.C., Gapstur S.M., Thun M.J. (2012). Daily aspirin use and cancer mortality in a large US cohort. J. Natl. Cancer Inst..

[84] Algra A.M., Rothwell P.M. (2012). Effects of regular aspirin on long-term cancer incidence and metastasis: A systematic comparison of evidence from observational studies versus randomised trials. Lancet Oncol..

[85] Rothwell P.M., Wilson M., Price J.F., Belch J.F., Meade T.W., Mehta Z. (2012). Effect of daily aspirin on risk of cancer metastasis: A study of incident cancers during randomised controlled trials. Lancet.

[86] Rothwell P.M., Price J.F., Fowkes F.G., Zanchetti A., Roncaglioni M.C., Tognoni G., Lee R., Belch J.F., Wilson M., Mehta Z. (2012). Short-term effects of daily aspirin on cancer incidence, mortality, and non-vascular death: Analysis of the time course of risks and benefits in 51 randomised controlled trials. Lancet.

[87] Elwood P.C., Morgan G., Pickering J.E., Galante J., Weightman A.L., Morris D., Kelson M., Dolwani S. (2016). Aspirin in the treatment of cancer: Reductions in metastatic spread and in mortality: A systematic review and meta-analyses of published studies. PLoS ONE.

[88] Rothwell P.M., Wilson M., Elwin C.E., Norrving B., Algra A., Warlow C.P., Meade T.W. (2010). Long-term effect of aspirin on colorectal cancer incidence and mortality: 20-year follow-up of five randomised trials. Lancet.

[89] Boutaud O., Sosa I.R., Amin T., Oram D., Adler D., Hwang H.S., Crews B.C., Milne G., Harris B.K., Hoeksema M., Knollmann B.C., Lammers P.E., Marnett L.J., Massion P.P., Oates J.A. (2016). Inhibition of the biosynthesis of prostaglandin E2 by low-dose aspirin: Implications for adenocarcinoma metastasis. Cancer Prev. Res..

[90] Wang Z., Nakayama T. (2010). Inflammation, a link between obesity and cardiovascular disease. Mediat. Inflamm..

[91] Mikawa S., Ohta Y., Kaji N., Islam M.S., Murata T., Ozaki H., Hori M. (2015). Time-dependent changes in inhibitory action of lipopolysaccharide on intestinal motility in rat. J. Vet. Med. Sci..

